# Association of Alcohol Consumption With Fat Deposition in a Community-Based Sample of Japanese Men: The Shiga Epidemiological Study of Subclinical Atherosclerosis (SESSA)

**DOI:** 10.2188/jea.JE20170191

**Published:** 2019-06-05

**Authors:** Masaki Sumi, Takashi Hisamatsu, Akira Fujiyoshi, Aya Kadota, Naoko Miyagawa, Keiko Kondo, Sayaka Kadowaki, Sentaro Suzuki, Sayuki Torii, Maryam Zaid, Atsushi Sato, Hisatomi Arima, Tomohiro Terada, Katsuyuki Miura, Hirotsugu Ueshima

**Affiliations:** 1 Department of Public Health, Shiga University of Medical Science, Shiga, Japan; 2 Department of Pharmacy, Shiga University of Medical Science Hospital, Shiga, Japan; 3 Department of Environmental Medicine and Public Health, Faculty of Medicine, Shimane University, Shimane, Japan; 4 Center for Epidemiologic Research in Asia, Shiga University of Medical Science, Shiga, Japan; 5 Department of Preventive Medicine and Public Health, Faculty of Medicine, Fukuoka University, Fukuoka, Japan

**Keywords:** alcohol consumption, visceral adipose tissue, fat deposition, anthropometric obesity indice

## Abstract

**Background:**

Excessive alcohol intake has been shown to be associated with cardiovascular disease via metabolic pathways. However, the relationship between alcohol intake and obesity has not been fully elucidated. We aimed to examine the association of alcohol consumption with fat deposition and anthropometric measures.

**Methods:**

From 2006–2008, we conducted a cross-sectional study in a population-based sample of Japanese men aged 40 through 79 years. Areas of abdominal visceral adipose tissue (VAT) and subcutaneous adipose tissue (SAT) were calculated using computed tomography imaging. Based on a questionnaire, we classified participants into five groups according to weekly alcohol consumption, excluding former drinkers: non-drinkers (0 g/week), 0.1–160.9, 161–321.9, 322–482.9, and ≥483 g/week. Multivariable linear regression was used to estimate adjusted means of obesity indices for each group.

**Results:**

We analyzed 998 men (mean age and body mass index [BMI], 63.8 years and 23.6 kg/m^2^, respectively). Higher weekly alcohol consumption was strongly and significantly associated with higher abdominal VAT area, percentage of VAT, and VAT-to-SAT ratio (all *P* for trend <0.001), and also with waist circumferences and waist-to-hip ratio (*P* for trend = 0.042 and 0.007, respectively). These associations remained significant after further adjustment for BMI, whereas alcohol consumption had no significant association with abdominal SAT area.

**Conclusions:**

Higher alcohol consumption was associated with higher VAT area, VAT%, and VAT-to-SAT ratio, independent of confounders, including BMI, in general Japanese men. These results suggest that alcohol consumption may have a potential adverse effect on visceral fat deposition.

## INTRODUCTION

Overweight and obesity are estimated to cause 3.4 million deaths globally each year,^1^ and the prevalence of obesity has increased worldwide.^2^ Obesity has a major impact on cardiovascular health.^3^^,^^4^ In addition, fat deposition has been recognized as an important risk factor for cardiovascular disease (CVD)^5^^,^^6^ and is related to metabolic syndrome^7^^–^^9^ and subclinical atherosclerosis.^8^^,^^10^ Specifically, visceral adipose tissue (VAT) showed a stronger association with CVD than subcutaneous adipose tissue (SAT).^5^ Moreover, greater VAT-to-SAT ratio (VSR) was also reported to be associated with CVD.^11^

Numerous epidemiological findings indicate that large amounts of alcohol intake are associated with a higher risk of CVD^12^^,^^13^ via metabolic pathways related to increased blood pressure,^14^ decreased glucose tolerability,^15^ and deteriorated lipid metabolism.^16^ However, limited evidence is available on how alcohol intake affects fat deposition, such as abdominal VAT and SAT. Only a few population-based studies investigated the association of alcohol consumption with VAT and SAT in Western countries,^17^ where BMI is generally higher than in other regions, including Japan. Other studies were also based on specific populations, such as overweight people,^18^ company employees,^19^ and hospital-based participants who underwent a health check-up.^20^ In addition, among all previous studies, former drinkers were not distinguished from lifetime abstainers, which could potentially have caused bias due to cessation from illness due to excessive alcohol intake. Furthermore, although a number of studies have investigated the relationships between alcohol intake and anthropometric measures, including body mass index (BMI),^21^^–^^25^ waist circumferences (WC),^16^^,^^22^^,^^23^^,^^25^^,^^26^ waist-to-height ratio (WHtR), or waist-to-hip ratio (WHR),^22^^,^^27^^–^^29^ these results remain inconsistent.

Therefore, we aimed to examine cross-sectional associations of alcohol consumption with measures of abdominal fat deposition, including computed tomography (CT)-based assessments of VAT and SAT and anthropometric indices, in a population-based sample of Japanese men, excluding former drinkers.

## METHODS

### Study participants and measurements

The Shiga Epidemiological Study of Subclinical Atherosclerosis (SESSA) is an ongoing prospective, population-based study of a random sample from a general Japanese population, as described elsewhere.^30^^,^^31^ Participants eligible for the present study were 1,094 men aged 40 through 79 years (mean 63.8; standard deviation [SD], 9.8 years) enrolled at baseline (May 2006–March 2008) in SESSA. After excluding 60 participants who were former drinkers and 36 participants with missing information, a total of 998 participants were analyzed in the present study. Of the 998 participants, 952 measured hip circumferences to calculate WHR. The present study was approved by the Institutional Review Board of Shiga University of Medical Science (Otsu, Japan), and all participants provided written informed consent.

A self-administered questionnaire was used to obtain information on demography, alcohol drinking, smoking habits, physical activity, medication use, medical history, and other lifestyle factors. After the participants completed the questionnaires, trained nurses confirmed answers to the questionnaire with the participants. Based on the questionnaire, the frequency of alcohol consumption during a typical week or month and the total alcohol intake on each occasion were determined and used to calculate the alcohol intake per week.^32^ Specifically, weekly intake of alcohol was assessed in units of “go” (a traditional Japanese unit of volume corresponding to 23 g of ethanol, that is, 7 go, 14 go, and 21 go correspond to 161 g, 322 g, and 483 g of ethanol, respectively), and was converted to grams of ethanol per week. One go is equivalent to 180 mL of Sake (Japanese rice wine) and corresponds to one bottle (500 mL) of beer, two single shots (75 mL) of whisky, or two glasses (180 mL) of wine. Participants who reported consuming 0.1 g/day or more of ethanol were regarded as drinkers.

Body weight and height were measured while the participants were wearing light clothing without shoes. Circumferences of waist and hip were measured twice at the levels of the umbilicus and maximal protrusion of the hip, respectively, in the end-exhalation phase while the participant was standing upright. The mean of the two measurements was used for analysis. BMI, WHtR, and WHR were calculated as weight (kg) divided by height squared (m^2^), WC (cm) divided by height (cm), and WC (cm) divided by hip circumference (cm), respectively. Blood pressure was measured twice consecutively in the right arm of the seated participant, after sitting quietly for 5 min, using an automated sphygmomanometer (BP-8800; Omron Health Care Co. Ltd, Tokyo, Japan). The mean of these two measurements was used for analyses.

Blood specimens were obtained early in the clinic visit, after fasting for at least at 12 h, and used for laboratory testing. Serum lipid concentrations were determined at a single laboratory (Shiga Laboratory; MEDIC, Shiga, Japan) that had been certified for standardized lipid measurements according to the protocols of the United States Centers for Disease Control and Prevention/Cholesterol Reference Method Laboratory Network. Total cholesterol and triglycerides were measured using enzymatic assays, and high-density lipoprotein cholesterol (HDL-C) was determined using a direct method. Plasma glucose levels were determined from NaF-treated plasma using a hexokinase glucose-6 phosphate-dehydrogenase enzymatic assay. Aminotransferase and Gamma-glutamyl transferase were measured using modified Japan Society of Clinical Chemistry reference method. HDL-C and Gamma-glutamyl transferase are known as biomarkers used to validate alcohol consumption.^33^^,^^34^ Smoking status was categorized into three groups: current, former, and never smokers. Participants who smoked in the last 30 days were defined as current smokers, whereas participants who had never smoked before were defined as never smokers, and smokers were queried for the average number of cigarettes smoked each day. Step count was recorded over 7 consequent days using a pedometer (DIGI-WALKER DW-200; Yamasa Tokei Keiki Co. Ltd, Tokyo, Japan). Then, we calculated the average steps per day.

### Abdominal adipose tissue areas

Areas of VAT and SAT were assessed using CT, as previously described.^35^ Abdominal VAT was defined as the fat enclosed by the inner aspect of the abdominal wall. Abdominal SAT was defined as the fat outside the outer aspects of the abdominal wall, but not including that fat located within the muscular fascia. While participants were supine, serial CT images were obtained using a protocol similar to one described previously.^36^ A single CT image of the L4–L5 vertebral space was selected to estimate areas of VAT and SAT. Adipose tissue was identified as showing attenuation between −190 and −30 Hounsfield units combined with anatomical interpretation by a reader. Studies of human cadavers have shown that the area measured by CT offers an accurate estimate of abdominal VAT,^37^ and the same or similar ranges of attenuation have been adopted to estimate VAT/SAT in population studies.^10^^,^^38^^–^^40^ The inner and outer aspects of the abdominal walls were manually tracked, and respective areas were calculated using image analysis software (SliceOmatic; Tomovision, Montreal, Canada). Two types of CT scanner were used during the examination period: a GE-Imatron C150 Electron Beam Tomography system (EBCT; GE Medical Systems, South San Francisco, CA, USA; slice thickness, 6 mm) for participants examined from May 2006 through August 2007 and a 16-row multidetector row CT system (MDCT, Aquilion-16™; Toshiba Medical Systems, Tochigi, Japan; slice thickness, 7 mm) for participants examined thereafter. All CT images were analyzed at Shiga University of Medical Science by a trained physician-researcher who was blinded to participant characteristics. Abdominal total adipose tissue (TAT) area, percentage of VAT (VAT%), and VSR were calculated as sum of VAT area (cm^2^) and SAT area (cm^2^), VAT area (cm^2^) divided by TAT area (cm^2^) and multiplied by 100, and VAT area (cm^2^) divided by SAT area (cm^2^), respectively.

### Statistical analysis

The participants were classified into five groups according to weekly alcohol intake: non-drinkers (0 g/week), 0.1–160.9, 161–321.9, 322–482.9, and ≥483 g/week. *P* values for trend across groups were determined either using linear regression when a response variable is continuous, or using logistic regression when it is categorical. An age-adjusted Pearson’s correlation was used to describe the relationship among obesity indices including anthropometric measures (BMI, WC, WHtR, and WHR) and abdominal fat deposition (VAT, SAT, TAT, VAT%, and VSR). A dose-response relationship between daily alcohol consumption and obesity measures was investigated using analysis of covariance. Adjusted covariates were age, daily cigarettes, daily steps, history of CVD, and history of liver disease. Dietary data were not available in our study. Because BMI is commonly used as an index of obesity and results from an imbalance between energy intake and expenditure, we also took this index into account in the adjusted model as a surrogate measure of energy intake by combined use with daily steps as energy expenditure. Further adjustment for use of any medication, such as glucocorticoid, anti-hyperlipidemic agents, and anti-hyperglycemic agents, did not substantially affect the findings, so these variables were not included in the model. Sensitivity analyses were performed in 926 participants without liver disease history and in 912 participants without CVD history. Analysis was performed using SPSS (version 22.0; SPSS, Inc., Chicago, IL, USA). Two-tailed *P* values of <0.05 were considered statistically significant.

## RESULTS

Demographics of 998 study participants based on alcohol intake are shown in Table 1. The mean BMI and weekly alcohol consumption was 23.6 kg/m^2^ and 173.4 g/week, respectively. Participants with higher alcohol intake were younger; taller; had higher systolic and diastolic blood pressure, HDL-C, triglyceride, fasting glucose, gamma-glutamyl transferase, transaminase, and cigarettes consumption; had a higher prevalence of current smoker; had a lower level of total cholesterol; and had a lower prevalence of CVD history. Among obesity measures, weekly alcohol consumption was positively associated with abdominal VAT, TAT, VAT%, VSR, WC, and WHR.

**Table 1.** Study sample characteristics according to weekly alcohol consumption in 998 men aged 40–79 years (SESSA, Shiga, Japan, 2006–2008)

*n*	Categories of weekly alcohol consumption (g/week)					Trend *P*

	Non-drinkers	0.1–160.9	161–321.9	322–482.9	≥483	

	192	378	226	129	73	
Age, years	65.4 (10.2)	63.4 (10.5)	64.4 (9.5)	62.6 (8.3)	61.2 (8.2)	0.004
Height, cm	165.9 (5.9)	166.5 (6.1)	166.3 (5.9)	167.2 (5.9)	167.5 (4.8)	0.026
Weight, kg	64.5 (9.5)	65.4 (9.8)	65.3 (9.8)	67.0 (10.2)	66.1 (9.3)	0.060
BMI, kg/m^2^	23.4 (2.9)	23.5 (3.0)	23.6 (2.9)	23.9 (3.1)	23.5 (3.1)	0.335
VAT area, cm^2^	108.3 (48.4)	117.5 (55.0)	120.5 (55.2)	132.0 (59.9)	125.9 (55.6)	<0.001
SAT area, cm^2^	120.2 (54.1)	124.1 (53.2)	119.2 (49.9)	125.1 (55.0)	114.5 (51.1)	0.615
TAT area, cm^2^	228.5 (94.0)	241.6 (98.6)	239.7 (96.4)	257.1 (104.5)	240.3 (96.1)	0.076
VAT%	47.4 (8.9)	48.2 (8.7)	49.7 (7.9)	51.2 (9.3)	52.0 (9.0)	<0.001
VSR	0.96 (0.36)	0.99 (0.37)	1.04 (0.35)	1.12 (0.42)	1.16 (0.44)	<0.001
WC, cm	84.4 (8.1)	85.3 (8.1)	85.9 (7.9)	86.2 (8.0)	85.7 (8.0)	0.045
WHtR	0.51 (0.05)	0.51 (0.05)	0.52 (0.05)	0.52 (0.05)	0.51 (0.05)	0.249
WHR^a^	0.92 (0.06)	0.92 (0.05)	0.93 (0.05)	0.93 (0.06)	0.92 (0.05)	0.015
Blood pressure, mm Hg						
Systolic	135.3 (18.3)	133.7 (17.6)	136.8 (18.9)	140.9 (19.9)	146.0 (21.6)	<0.001
Diastolic	77.3 (10.2)	78.6 (10.5)	80.7 (10.6)	82.7 (12.2)	85.5 (10.9)	<0.001
Total-cholesterol, mg/dL	210.0 (33.7)	208.7 (31.6)	207.6 (34.2)	201.4 (32.5)	204.7 (35.6)	0.029
HDL-C, mg/dL	53.2 (13.5)	57.9 (15.7)	61.2 (17.8)	61.4 (15.8)	66.7 (23.4)	<0.001
Triglycerides, mg/dL	124.2 (66.0)	119.8 (68.6)	126.8 (82.1)	142.5 (124.6)	151.8 (81.6)	0.001
Fasting glucose, mg/dL	101.0 (17.3)	101.2 (21.1)	105.5 (24.1)	103.4 (18.7)	105.8 (25.2)	0.022
Gamma-glutamyl transferase, U/L	38.0 (42.4)	40.2 (35.6)	63.1 (74.7)	85.6 (73.8)	136.9 (175.0)	<0.001
Alanine aminotransferase, U/L	26.1 (15.9)	25.0 (14.4)	26.7 (18.2)	26.2 (14.8)	35.3 (28.9)	0.002
Aspartate aminotransferase, U/L	25.6 (8.7)	25.1 (8.4)	29.2 (23.0)	29.2 (12.4)	40.0 (32.3)	<0.001
Daily steps	7297 (2981)	8036 (3192)	8257 (3372)	8154 (3071)	7728 (3373)	0.078
Daily number of cigarettes	6.9 (11.0)	5.1 (9.8)	6.6 (10.4)	8.7 (11.7)	10.4 (14.9)	0.001
Smoking, %						
Current	33.3	26.5	34.5	41.9	41.1	0.007
Former	42.7	51.6	53.5	52.7	46.6	0.179
History of CVD, %	14.6	7.1	7.5	7.0	6.8	0.035
History of liver disease, %	6.3	5.3	9.3	9.3	9.6	0.272

BMI, body mass index; CVD, cardiovascular disease; HDL-C, high density lipoprotein cholesterol; SAT, abdominal subcutaneous adipose tissue; SESSA, Shiga Epidemiological Study of Subclinical Atherosclerosis; SD, standard deviation; TAT, abdominal total adipose tissue; VAT, abdominal visceral adipose tissue; VAT%, percentage of visceral adipose tissue; VSR, VAT-SAT ratio; WC, waist circumference; WHR, waist-to-hip ratio; WHtR, waist-to-height ratio.

Values are expressed as mean (standard deviation), or percentage.

*P* values for trend were obtained using linear regression (for continuous variables) or logistic regression (for categorical variables).

^a^A total of 952 (non-drinkers, 184; 0.1–160.9 g/week, 368; 161–321.9 g/week, 213; 322–482.9 g/week, 119; ≥483 g/week, 68) men underwent measurement of hip circumferences.

According to age-adjusted Pearson’s correlation coefficients (*r*) between obesity indices (eTable 1), BMI, WC, and WHtR were strongly and positively correlated with abdominal TAT, VAT, and SAT area (all correlation coefficients range from 0.72 to 0.88), but not with VAT% or VSR (all correlation coefficients range from −0.05 to 0.04). WHR was strongly and positively correlated with abdominal TAT, VAT, and SAT area (correlation coefficients, 0.88, 0.67, and 0.63, respectively) and was weakly with VAT% and VSR (correlation coefficients, 0.13 and 0.12, respectively). Abdominal VAT area was positively correlated with abdominal SAT area, TAT area, VAT%, and VSR (correlation coefficients, 0.66, 0.92, 0.47 and 0.45, respectively), whereas abdominal VAT% and VSR were weakly but negatively correlated with abdominal SAT (correlation coefficients, −0.28 and −0.29, respectively).

Adjusted means of obesity indices are shown according to weekly alcohol consumption in Table 2. Higher weekly alcohol consumption was strongly and significantly associated with higher abdominal VAT area, VAT%, and VSR (all *P* for trend <0.001), and also with anthropometric indices such as WC and WHR (*P* for trend = 0.042 and 0.007, respectively). These associations also persisted after further adjustment for BMI (Table 3). On the other hand, there was no significant association between weekly alcohol consumption and abdominal SAT area (Table 2 and Table 3). In the sensitivity analyses, similar results were observed among participants without liver disease history (eTable 2) and CVD history (eTable 3).

**Table 2.** Adjusted mean values^a^ of obesity indices according to weekly alcohol consumption in 998 men aged 40–79 years (SESSA, Shiga, Japan, 2006–2008)

*n*	Categories of weekly alcohol consumption (g/week)					Trend *P*

	Non-drinkers	0.1–160.9	161–321.9	322–482.9	≥483	

	192	378	226	129	73	
VAT area, cm^2^	105.3 (3.9)	117.7 (2.7)	122.2 (3.5)	133.2 (4.7)	125.3 (6.2)	<0.001
SAT area, cm^2^	118.7 (3.7)	123.8 (2.6)	121.0 (3.4)	125.8 (4.5)	112.8 (6.0)	0.360
TAT area, cm^2^	224.0 (6.9)	241.5 (4.9)	243.3 (6.3)	259.1 (8.3)	238.1 (11.1)	0.028
VAT%	47.2 (0.6)	48.3 (0.4)	49.7 (0.6)	51.2 (0.8)	52.1 (1.0)	<0.001
VSR	0.95 (0.03)	0.99 (0.02)	1.04 (0.02)	1.13 (0.03)	1.17 (0.04)	<0.001
BMI, kg/m^2^	23.3 (0.2)	23.5 (0.2)	23.7 (0.2)	23.9 (0.3)	23.4 (0.3)	0.489
WC, cm	84.0 (0.6)	85.4 (0.4)	86.2 (0.5)	86.3 (0.7)	85.4 (0.9)	0.042
WHtR	0.51 (0.00)	0.51 (0.00)	0.52 (0.00)	0.52 (0.00)	0.51 (0.01)	0.066
WHR^b^	0.91 (0.00)	0.92 (0.00)	0.93 (0.00)	0.93 (0.00)	0.93 (0.01)	0.007

BMI, body mass index; CVD, cardiovascular disease; SAT, abdominal subcutaneous adipose tissue; SE, standard error; SESSA, Shiga Epidemiological Study of Subclinical Atherosclerosis; TAT, abdominal total adipose tissue; VAT, abdominal visceral adipose tissue; VAT%, percentage of visceral adipose tissue; VSR, VAT-SAT ratio; WC, waist circumference; WHR, waist-to-hip ratio; WHtR, waist-to-height ratio.

Values are expressed as mean (SE). *P* values for trend were obtained using linear regression.

^a^Mean values were adjusted using analysis of covariance for age, daily steps, daily number of cigarettes, history of CVD, and history of liver disease.

^b^A total of 952 (non-drinkers, 184; 0.1–160.9 g/week, 368; 161–321.9 g/week, 213; 322–482.9 g/week, 119; ≥483 g/week, 68) men underwent measurement of hip circumferences.

**Table 3.** Adjusted mean values^a^ of abdominal fat deposition indices according to weekly alcohol consumption in 998 men aged 40–79 years after further adjustment for BMI (SESSA, Shiga, Japan, 2006–2008)

*n*	Categories of weekly alcohol consumption (g/week)					Trend *P*

	Non-drinkers	0.1–160.9	161–321.9	322–482.9	≥483	

	192	378	226	129	73	
VAT area, cm^2^	108.2 (2.8)	118.1 (2.0)	121.1 (2.5)	128.7 (3.3)	127.1 (4.4)	<0.001
SAT area, cm^2^	121.9 (2.2)	124.3 (1.6)	119.8 (2.0)	120.8 (2.7)	114.8 (3.6)	0.112
TAT area, cm^2^	230.1 (3.8)	242.4 (2.7)	240.9 (3.5)	249.5 (4.7)	241.8 (6.2)	0.021
VAT%	47.2 (0.6)	48.3 (0.4)	49.7 (0.5)	51.3 (0.8)	52.1 (1.0)	<0.001
VSR	0.95 (0.03)	1.00 (0.02)	1.04 (0.02)	1.13 (0.03)	1.17 (0.04)	<0.001
WC, cm	84.5 (0.3)	85.5 (0.2)	86.0 (0.3)	85.5 (0.4)	85.8 (0.5)	0.006
WHtR	0.51 (0.00)	0.51 (0.00)	0.52 (0.00)	0.51 (0.00)	0.52 (0.00)	0.004
WHR^b^	0.92 (0.00)	0.92 (0.00)	0.93 (0.00)	0.93 (0.00)	0.93 (0.00)	0.009

BMI, body mass index; CVD, cardiovascular disease; SAT, abdominal subcutaneous adipose tissue; SE, standard error; SESSA, Shiga Epidemiological Study of Subclinical Atherosclerosis; TAT, abdominal total adipose tissue; VAT, abdominal visceral adipose tissue; VAT%, percentage of visceral adipose tissue; VSR, VAT-SAT ratio; WC, waist circumference; WHR, waist-to-hip ratio; WHtR, waist-to-height ratio.

Values are expressed as mean (SE). *P* values for trend were obtained using linear regression.

^a^Mean values were adjusted for BMI in addition to age, daily steps, daily number of cigarettes, history of CVD, and history of liver disease.

^b^A total of 952 (non-drinkers, 184; 0.1–160.9 g/week, 368; 161–321.9 g/week, 213; 322–482.9 g/week, 119; ≥483 g/week, 68) men underwent measurement of hip circumferences.

## DISCUSSION

### Principal findings

In this population-based study in Asia, with one of the lowest levels of BMI among the relevant studies, we cross-sectionally observed significant dose-dependent associations between weekly alcohol consumption and abdominal VAT area, VAT%, VSR, TAT, WC, and WHR; these associations were independent of age, daily steps, smoking, CVD history, and history of liver disease. These associations were still significant after further adjustment for BMI, as a surrogate measure of energy intake. In contrast, we observed no significant association between alcohol intake and abdominal SAT area.

### In the context of current literature

Our results support past demonstrations with respect to the positive association between alcohol consumption and VAT-related indices. Molenaar et al, in 2,926 participants from the Framingham Heart Study, with mean age of 49.5 years and mean BMI of 27.7 kg/m^2^, revealed that a large amount of alcohol was associated with a higher abdominal VAT volume but not with SAT volume in men, whereas, in women, higher alcohol intake was associated with lower abdominal SAT volume but not with VAT volume.^17^ Among 87 apparently healthy Italian women aged only 38 years with a mean BMI of 24.2 kg/m^2^, a positive association was found between alcohol intake and VAT area.^41^ For previous results based on specific populations, in a hospital-based study of 951 Korean men who received a health check-up, with mean age of 52.4 years and mean BMI of 25.0 kg/m^2^, alcohol consumption was associated with higher abdominal VAT area and lower abdominal SAT area.^20^ A significant relationship of higher alcohol intake and VAT area has also been previously confirmed in Japanese male employees^19^ and in Japanese overweight men.^18^ However, among all previous studies, former drinkers were not distinguished from lifetime abstainers. Our findings of a higher alcohol intake related to abdominal VAT area, VAT%, and VSR among Japanese general men without former drinkers, in addition to these pieces of past evidence, indicate that alcohol consumption is positively associated with VAT indices, although the relationship between alcohol consumption and SAT is equivocal.

The effects of alcohol consumption on anthropometrical indicators remain controversial. Similar to our findings, no significant relationship between alcohol consumption and BMI were reported in Japanese men aged 21–65 years,^18^ whereas alcohol consumption was inversely or J-shaped associated with BMI in other studies of American men and women^21^^,^^24^ and Japanese men,^25^ and WC in Japanese men^25^ and American^16^ and Danish^26^ men and women with middle and older age. On the other hand, positive associations between alcohol intake and BMI were found in British and Korean with middle to older age.^22^^,^^23^ Consistent with our results, the amount of alcohol was positively related to WC,^22^^,^^23^ and to WHR in the United States^27^ and Europe.^22^^,^^28^^,^^29^ Based on these findings, the influence of alcohol on anthropometric obesity measures remains unclear. These inconsistencies between studies may be because of the difference in the study sample demographics, such as obesity distribution and alcohol intake, and measurement techniques for anthropometric and alcohol measures. Despite these inconsistencies, the positive association between alcohol consumption and VAT indices has been robustly coincided with previous studies, and VAT indices were more strongly associated with metabolic risk factor clustering,^42^ atherosclerosis,^43^ and CVD^5^ compared with anthropometric indices.

### Potential mechanisms

The exact mechanism of the association between alcohol intake and abdominal fat deposition remains unclear, but several speculations have been made. Alcohol intake concomitant with meal contribute to VAT accumulation through decreased VAT suppressors, such as leptin and glucagon-like peptide 1 (GLP-1).^44^^–^^47^ Alcohol intake also increases the plasma cortisol level and decreases serum testosterone-to-cortisol ratio, the alterations of which promote VAT accumulation.^48^^–^^51^ Further, higher alcohol intake was presumed to reduce serum adiponectin levels,^52^ which were inversely associated with VAT accumulation.^53^ It is noteworthy that leptin, cortisol, testosterone, and adiponectin were reported to be more strongly associated with VAT accumulation than with SAT accumulation.^46^^,^^49^^,^^51^^,^^53^ Additionally, hypertriglyceridemia induced via alcohol intake is thought to be a result of increased very-low-density lipoprotein secretion, impaired lipolysis, and increased free fatty acid fluxes from adipose tissue to the liver.^45^^,^^54^ VAT is also correlated with serum triglycerides levels.^55^ Thus, it is speculated that alcohol consumption affects metabolic balance susceptible to fat accumulation around mesenteric and liver. Meanwhile, alcohol intake is potentially relevant to dietary habit. Although we did not have detailed dietary records, positive relationships were observed of alcohol consumption with energy intake^18^ and dietary fat,^56^ which were positively associated with VAT.^18^^,^^57^ However, no significant change was observed in the relationship between alcohol consumption and VAT indices when we add BMI to the adjustment variables as a surrogate measure of energy intake. Further investigation is necessary to confirm this association in other population and identify the potential mechanism.

### Strengths and limitations

Our study has several limitations. First, the study design was cross-sectional. Therefore, causal and longitudinal relationships were not addressed. Second, data on alcohol intake were based on self-report, leading to potential misclassification. However, our observation of positive association of alcohol consumption with HDL-C and Gamma-glutamyl transferase validate the classification. Third, there is possibility that our findings may, in part, be explained by differences in unknown or residual confounders, such as dietary habits. Finally, because only Japanese men were included for analyses, our results are restricted to men of a single ethnic group. However, population homogeneity reduces possible confounding from cultural, environmental, and genetic variation. Strength of our study include the general population-based design, use of a standardized protocol in assessing outcomes, such as CT-based abdominal VAT and SAT area, other adjusting covariates, and the exclusion of former drinkers for the analysis, which minimized bias due to cessation because of illness caused by alcohol.

### Conclusions

The present study demonstrated that higher alcohol consumption was associated with a greater degree of abdominal VAT area, VAT%, and VSR in a population-based sample of Japanese men, independent of possible confounding factors, including BMI. Our findings suggest that alcohol consumption may potentially and adversely affect fat deposition.

## APPENDIX A. SUPPLEMENTARY DATA

eTable 1. Age-adjusted Pearson’s correlation coefficients among obesity indices in 998 men aged 40–79 years (SESSA, Shiga, Japan, 2006–2008)

eTable 2. Adjusted mean values^a^ of abdominal fat deposition indices according to weekly alcohol consumption in 926 men aged 40–79 years without history of liver disease (SESSA, Shiga, Japan, 2006–2008)

eTable 3. Adjusted mean values^a^ of obesity indices according to weekly alcohol consumption in 912 men aged 40–79 years without CVD history (SESSA, Shiga, Japan, 2006–2008)

## APPENDIX B.

### Members of the SESSA Research Group

Chairperson: Hirotsugu Ueshima (Center for Epidemiologic Research in Asia, Department of Public Health, Shiga University of Medical Science, Otsu, Shiga).

Co-chairpersons: Katsuyuki Miura (Department of Public Health, Shiga University of Medical Science, Otsu, Shiga).

Research members: Minoru Horie, Yasutaka Nakano, Takashi Yamamoto, Hideki Hayashi (Department of Cardiovascular and Respiratory Medicine, Shiga University of Medical Science, Otsu, Shiga), Emiko Ogawa (Health Administration Center, Shiga University of Medical Science, Otsu, Shiga), Hiroshi Maegawa, Itsuko Miyazawa (Division of Endocrinology and Metabolism, Department of Medicine, Shiga University of Medical Science, Otsu, Shiga), Kiyoshi Murata (Department of Radiology, Shiga University of Medical Science, Otsu, Shiga), Kenichi Mitsunami (Vories Memorial Hospital, Omihachiman, Shiga), Kazuhiko Nozaki (Department of Neurosurgery, Shiga University of Medical Science, Otsu, Shiga), Ikuo Tooyama, Akihiko Shiino (Molecular Neuroscience Research Center, Shiga University of Medical Science, Otsu, Shiga), Teruhiko Tsuru (Department of Urology, Shiga University of Medical Science, Otsu, Shiga), Hisakazu Ogita, Akio Shimizu (Division of Medical Biochemistry, Department of Biochemistry and Molecular Biology, Shiga University of Medical Science, Otsu, Shiga), Naomi Miyamatsu (Department of Clinical Nursing, Shiga University of Medical Science, Otsu, Shiga), Toru Kita (Kyoto University, Kyoto), Takeshi Kimura (Department of Cardiovascular Medicine, Kyoto University, Kyoto), Yoshihiko Nishio (Department of Diabetes, Metabolism, and Endocrinology, Kagoshima University, Kagoshima), Yasuyuki Nakamura (Department of Food Science and Human Nutrition, Faculty of Agriculture, Ryukoku University, Otsu, Shiga), Tomonori Okamura (Department of Preventive Medicine and Public Health, School of Medicine, Keio University, Tokyo), Akira Sekikawa, Emma JM Barinas-Mitchell (Department of Epidemiology, Graduate School of Public Health, University of Pittsburgh, Pittsburgh, PA, USA), Daniel Edmundowicz (Department of Medicine, Section of Cardiology, School of Medicine, Temple University, Philadelphia, PA, USA), Takayoshi Ohkubo (Department of Hygiene and Public Health, Teikyo University School of Medicine, Tokyo), Atsushi Hozawa (Department of Preventive Medicine and Epidemiology, Tohoku Medical Megabank Organization, Tohoku University, Sendai, Miyagi), Yoshitaka Murakami (Division of Medical Statistics, Department of Social Medicine, Toho University, Tokyo), Nagako Okuda (Department of Health and Nutrition, University of Human Arts and Sciences, Saitama), Hisatomi Arima (Department of Preventive Medicine and Public Health, Faculty of Medicine, Fukuoka University, Fukuoka), Yoshikuni Kita (Faculty of Nursing Science, Tsuruga Nursing University, Tsuruga, Fukui), Takashi Hisamatsu (Department of Environmental Medicine and Public Health, Faculty of Medicine, Shimane University, Izumo, Shimane), Aya Higashiyama (Research and Development Initiative Center, National Cerebral and Cardiovascular Center, Suita, Osaka), Shinya Nagasawa (Department of Epidemiology and Public Health, Kanazawa Medical University, Kanazawa, Ishikawa), Koichiro Azuma (Institute for Integrated Sports Medicine School of Medicine, Keio University, Tokyo), Masahiko Yanagita (Faculty of Health and Sports Science, Doshisha University, Kyoto), Robert D. Abbott, Aya Kadota, Seiko Ohno, Maryam Zaid (Center for Epidemiologic Research in Asia, Shiga University of Medical Science, Otsu, Shiga), Akira Fujiyoshi, Naoyuki Takashima, Takashi Kadowaki, Sayaka Kadowaki, Naoko Miyagawa, Keiko Kondo, Sayuki Torii, Yoshino Saito, Sentaro Suzuki and Takahiro Ito, Atsushi Satoh, Masahiro Yamazoe (Department of Public Health, Shiga University of Medical Science, Otsu, Shiga).
