# Supplementary material for: Association of Alcohol Consumption With Fat Deposition in a Community-Based Sample of Japanese Men: The Shiga Epidemiological Study of Subclinical Atherosclerosis (SESSA)
Source: J Epidemiol. 2019 Jun 5;29(6):205–12. doi: 10.2188/jea.JE20170191 (PMC6522392; doi:10.2188/jea.JE20170191)
Supplement: Supplementary file 1 [file je-29-205-s001.pdf]

eTable 1. Age-adjusted Pearson's correlation coefficients among obesity indices in 998 men aged 40–79 years (SESSA, Shiga, Japan, 2006–2008)

		BMI (kg/m^2)	WC (cm)	WHtR	WHR[a]	VAT area (cm^2)	SAT area (cm^2)	TAT area (cm^2)	VAT%
WC	Coefficient	0.87							
	P value	*P*<0.001							
WHtR	Coefficient	0.88	0.94						
	P value	*P*<0.001	*P*<0.001						
WHR[a]	Coefficient	0.62	0.83	0.84					
	P value	*P*<0.001	*P*<0.001	*P*<0.001					
VAT area	Coefficient	0.72	0.77	0.75	0.67				
	P value	*P*<0.001	*P*<0.001	*P*<0.001	*P*<0.001				
SAT area	Coefficient	0.82	0.83	0.81	0.63	0.66			
	P value	*P*<0.001	*P*<0.001	*P*<0.001	*P*<0.001	*P*<0.001			
TAT area	Coefficient	0.84	0.88	0.85	0.88	0.92	0.91		
	P value	*P*<0.001	*P*<0.001	*P*<0.001	*P*<0.001	*P*<0.001	*P*<0.001		
VAT%	Coefficient	-0.03	0.04	0.03	0.13	0.47	-0.28	0.11	
	P value	*P*=0.309	*P*=0.183	*P*=0.403	*P*<0.001	*P*<0.001	*P*<0.001	*P*<0.001	
VSR	Coefficient	-0.05	0.02	0.01	0.12	0.45	-0.29	0.10	0.97
	P value	*P*=0.122	*P*=0.493	*P*=0.737	*P*<0.001	*P*<0.001	*P*<0.001	*P*=0.002	*P*<0.001

BMI, body mass index; SAT, abdominal subcutaneous adipose tissue; SESSA, Shiga Epidemiological Study of Subclinical Atherosclerosis; TAT, abdominal total adipose tissue; VAT, abdominal visceral adipose tissue; VAT%, percentage of visceral adipose tissue; VSR, VAT-SAT ratio; WC, waist circumference; WHR, waist-to-hip ratio; WHtR, waist-to-height ratio.

[a] A total of 952 men underwent measurement of hip circumferences.

eTable 2. Adjusted mean values[a] of abdominal fat deposition indices according to weekly alcohol consumption in 926 men aged 40–79 years without history of liver disease (SESSA, Shiga, Japan, 2006–2008)

	Categories of weekly alcohol consumption (g/week)					
	Non-drinkers	0.1–160.9	161–321.9	322–482.9	≥483	Trend *P*
n	180	358	205	117	66	
VAT area (cm^2)	107.8 (2.8)	118.1 (2.0)	122.2 (2.6)	129.1 (3.5)	122.9 (4.7)	<0.001
SAT area (cm^2)	121.3 (2.3)	124.0 (1.6)	119.3 (2.1)	120.9 (2.8)	113.7 (3.7)	0.100
TAT area (cm^2)	229.1 (4.0)	242.0 (2.8)	241.5 (3.7)	250.0 (4.9)	236.5 (6.5)	0.014
VAT%	47.3 (0.7)	48.3 (0.5)	49.9 (0.6)	51.3 (0.8)	51.5 (1.1)	<0.001
VSR	0.95 (0.03)	1.00 (0.02)	1.05 (0.03)	1.13 (0.03)	1.14 (0.05)	<0.001
BMI (kg/m^2)	23.4 (0.2)	23.5 (0.2)	23.5 (0.2)	24.1 (0.3)	23.5 (0.4)	0.364
WC (cm)	84.5 (0.3)	85.4 (0.2)	85.9 (0.3)	85.5 (0.4)	85.5 (0.5)	0.010
WHtR	0.51 (0.00)	0.51 (0.00)	0.52 (0.00)	0.51 (0.00)	0.51 (0.00)	0.005
WHR[b]	0.92 (0.00)	0.92 (0.00)	0.93 (0.00)	0.93 (0.00)	0.92 (0.01)	0.024

BMI, body mass index; CVD, cardiovascular disease; SAT, abdominal subcutaneous adipose tissue; SE, standard error; SESSA, Shiga Epidemiological Study of Subclinical Atherosclerosis; TAT, abdominal total adipose tissue; VAT, abdominal visceral adipose tissue; VAT%, percentage of visceral adipose tissue; VSR, VAT-SAT ratio; WC, waist circumference; WHR, waist-to-hip ratio; WHtR, waist-to-height ratio.

Values are expressed as mean (SE). *P* values for trend were obtained using linear regression.

[a] Mean values were adjusted for BMI, age, daily steps, daily number of cigarettes, history of CVD, and history of liver disease.

[b] A total of 886 (non-drinkers, 172; 0.1–160.9 g/week, 349; 161–321.9 g/week, 195; 322–482.9 g/week, 107; ≥483 g/week, 63) men underwent measurement of hip circumferences.

eTable 3. Adjusted mean values[a] of obesity indices according to weekly alcohol consumption in 912 men aged 40–79 years without CVD history (SESSA, Shiga, Japan, 2006–2008)

	Categories of weekly alcohol consumption (g/week)					
	Non-drinkers	0.1–160.9	161–321.9	322–482.9	≥483	Trend *P*
n	164	351	209	120	68	
VAT area (cm^2)	108.3 (3.0)	116.6 (2.0)	119.1 (2.6)	127.3 (3.5)	122.4 (4.6)	<0.001
SAT area (cm^2)	120.7 (2.4)	123.5 (1.6)	119.8 (2.1)	119.6 (2.8)	113.9 (3.7)	0.159
TAT area (cm^2)	229.0 (4.2)	240.1 (2.8)	238.9 (3.7)	246.9 (4.8)	236.3 (6.4)	0.070
VAT%	47.5 (0.7)	48.1 (0.5)	49.4 (0.6)	51.2 (0.8)	51.6 (1.1)	<0.001
VSR	0.96 (0.03)	0.99 (0.02)	1.03 (0.03)	1.13 (0.03)	1.14 (0.05)	<0.001
WC (cm)	84.4 (0.3)	85.3 (0.2)	85.8 (0.3)	85.3 (0.4)	85.4 (0.5)	0.029
WHtR	0.51 (0.00)	0.51 (0.00)	0.52 (0.00)	0.51 (0.00)	0.51 (0.00)	0.007
WHR[b]	0.91 (0.00)	0.92 (0.00)	0.93 (0.00)	0.93 (0.00)	0.92 (0.00)	0.015

BMI, body mass index; CVD, cardiovascular disease; SAT, abdominal subcutaneous adipose tissue; SE, standard error; SESSA, Shiga Epidemiological Study of Subclinical Atherosclerosis; TAT, abdominal total adipose tissue; VAT, abdominal visceral adipose tissue; VAT%, percentage of visceral adipose tissue; VSR, VAT-SAT ratio; WC, waist circumference; WHR, waist-to-hip ratio; WHtR, waist-to-height ratio.

Values are expressed as mean (SE). *P* values for trend were obtained using linear regression.

[a] Mean values were adjusted using analysis of covariance for BMI, age, daily steps, daily number of cigarettes, and history of liver disease.

[b] A total of 867 (non-drinkers, 156; 0.1–160.9 g/week, 341; 161–321.9 g/week, 197; 322–482.9 g/week, 110; ≥483 g/week, 63) men underwent measurement of hip circumferences.
